# A novel medical device that combines filtered sunlight phototherapy and kangaroo care to treat neonatal jaundice: bench feasibility study

**DOI:** 10.1038/s41390-025-04559-z

**Published:** 2026-01-14

**Authors:** Daniel J. John, Stephen C. John, Tina Slusher

**Affiliations:** 1 University of Michigan Medical School, Ann Arbor, MI USA; 2 Department of Cardiology, University of Texas, Houston, TX USA; 3 Department of Pediatrics, University of Minnesota, Minneapolis, MN USA

## Abstract

**Background:**

Phototherapy, involving therapeutic blue light, is essential for treating neonatal jaundice/hyperbilirubinemia. Kangaroo care involves prolonged skin to skin contact between the infant and parent, especially important for newborn infants. These experiments seek to investigate the ability of a novel medical device, BiliRoo, to deliver filtered sunlight phototherapy in a kangaroo care carrier, to assess whether harmful UV-A (and by extension UV-B) was filtered out while adequate therapeutic blue light was allowed to pass through at multiple caregiver angles.

**Methods:**

In a bench model, the delivered UV-A radiation and therapeutic blue irradiance were measured for Air Blue and Gila Titanium filters from 10:00 a.m. to 8 p.m. at angles of 30°, 60°, and 90° from the ground.

**Results:**

The mean values of irradiance for both filters at all three angles were above the threshold of intensive phototherapy (30 μW/cm²/nm). For both filters, over 99% of the mean UV-A radiation was filtered out at all three angles.

**Conclusions:**

In a bench model, BiliRoo is able to filter harmful UV radiation while still delivering adequate average therapeutic blue light at a range of caregiver angles. Further testing on usability and clinical feasibility, safety and efficacy are warranted.

**Impact:**

- Introduces a novel non-electric medical device that combines filtered sunlight phototherapy and kangaroo care to treat infant jaundice.
- Demonstrates the ability of the device to provide adequate therapeutic blue irradiance to treat jaundice while blocking harmful UV sunlight for caregivers at multiple angles, enabling potential at-home and mobile care.

## Introduction

Worldwide, an estimated 80% of infants develop jaundice^1^ due to high levels of bilirubin in the blood. Causes of infant jaundice include an immature liver due to prematurity, sepsis, inadequate breastfeeding, deficient liver enzymes, maternal/infant blood group incompatibilities, Rhesus’s disease and/or Glucose-6-phosphate dehydrogenase deficiency a congenital enzyme deficiency. Typically, mild cases of jaundice resolve within 3–5 days without complications^2^. However, 8–11% of neonates develop moderate to severe jaundice^3^; If severe jaundice is left untreated, the free unconjugated bilirubin can pass through the blood-brain barrier and can lead to encephalopathy, cerebral palsy, deafness, and even death^4^.

Electric-powered phototherapy, the mainstay of treatment for jaundice in high-resource settings, has significantly reduced the need for exchange transfusions and brain damage from jaundice in those settings^2^. Unfortunately, due to the high costs of the machines, lack of continuous electricity, lack of replacement parts, and lack of enough trained personnel for implementation and maintenance, low- and middle-income settings have limited access to high quality phototherapy and consequently bear the largest burden of severe jaundice^2^. Every year, an estimated 6 million infants with severe jaundice in low-resource settings like Sub-Saharan Africa currently lack consistent access to treatment^5^.

Filtered sunlight phototherapy is a safe and effective means of providing phototherapy that has been validated in large randomized control trials^6^. The filter selectively allows therapeutic blue light from the sun to pass through while blocking harmful UV rays to provide simple, effective, non-electric phototherapy. While the currently used tent and room models have been shown to be effective delivery of therapeutic phototherapy, it is difficult for parents to accompany the babies and provide concurrent kangaroo care; they would be exposed and unable to do anything else while their infant is receiving therapy. Consequently, neonates are often separated from their parents and can overheat from the sun. Pediatric nurses who are already stretched thin with a high patient-to-nursing ratio have to remember to go out of the hospital to check the baby’s temperature hourly lest the baby develop hyper/hypothermia.

Current filtered sunlight phototherapy also lacks the essential kangaroo care aspect, in which the neonate has prolonged skin-to-skin contact with the parent^7^. The benefits associated with kangaroo care^8,9^ are not available using current methods of treatment with filtered phototherapy tents/rooms. These barriers as well as the inability for mothers and community health extension workers to effectively monitor these baby’s temperatures have prevented widespread implementation of an otherwise effective, low-cost, and non-electric treatment.

**Fig. 1:**
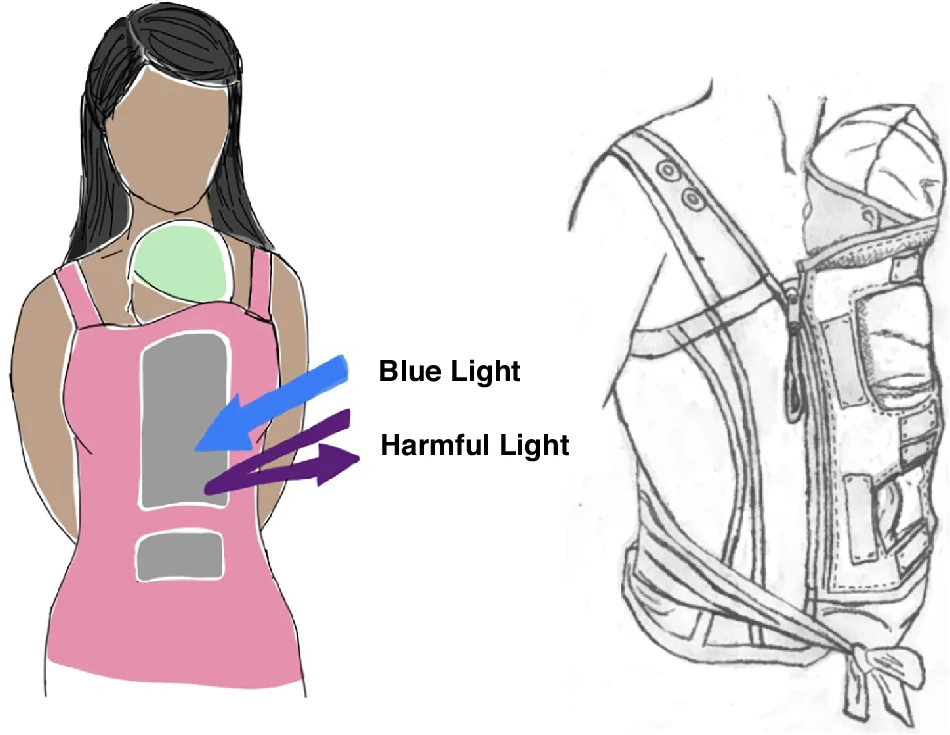
Schematic of BiliRoo Model. BiliRoo is a kangaroo care sling that integrates filtered sunlight phototherapy via a filter on the infant’s back to treat infant jaundice. Diagram was used with permission from www.biliroo.com.

A novel device, BiliRoo, that combines the safe, proven, and effective treatments of filtered sunlight phototherapy and kangaroo care has been developed and is shown below in Fig. 1. (See www.biliroo.com for additional information).

The objective of the study is to assess the effectiveness of this novel medical device in a bench model to deliver therapeutic blue light of >30 µW/cm^2^/nm threshold for at least 4 h a day while blocking at least 90% of harmful UVA radiation and not increasing the air temperature under the filter.

## Methods

**Fig. 2:**
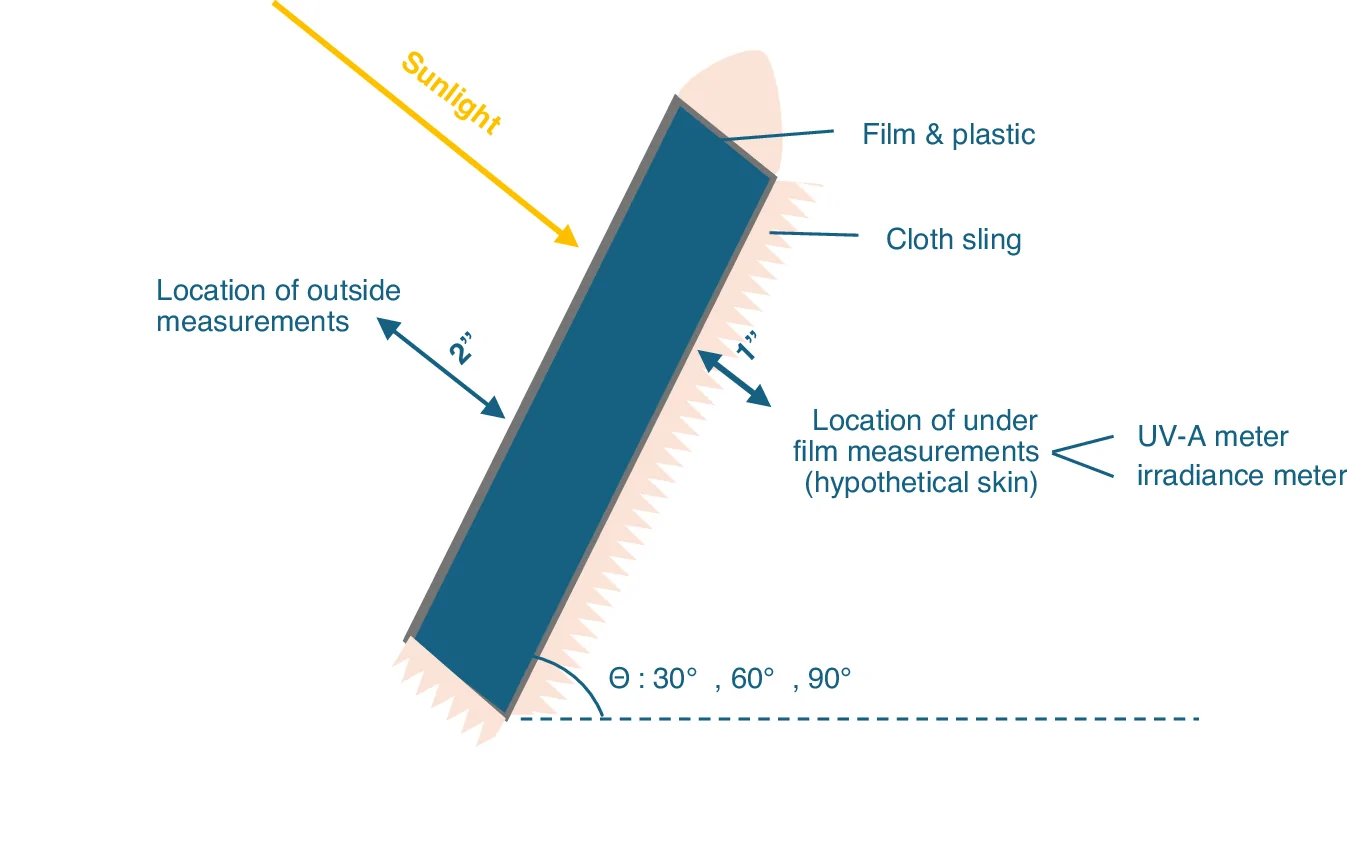
Experimental set-up*.* The set-up involved the cloth sling held towards the sun at different angles with the measurement tools (irradiance meter and UVA meter) a set distance from the filter.

The bench set up to assess for ability to filter out harmful UV-A irradiance but allow therapeutic blue light to pass through was modeled after Vreman et al.’s previous experiments^10^. See Fig. 2 for the experimental setup.

A metalized film [Gila Platinum (M)] and a metal oxynitride film [Air Blue 80 (S)] were utilized for the study, based on the results from Vreman et al.’s bench study on filtered sunlight phototherapy^10^. Both filters were reinforced with a clear plastic sheet for structural stability. The angle between the reinforced filters and the ground was varied from 30° to 90° in 30° increments to simulate the infant’s caregiver sitting at different angles. Tests were not performed at 0° as these tests have already been performed by Vreman et al.^10^.

UV-A radiation was measured in µW/cm^2^ using the Goldilux Radiometer/Photometer with a GAP-1 Probe (Oriel Instruments, Stratford, CT). Irradiance, measured in µW/cm^2^/nm was measured using the Bili-Blanket Meter II (C) (General Electric, Fairfield, CT) irradiance meter. Measurements of delivered therapeutic blue irradiance and harmful UV-A radiation were performed one inch behind the metalized filters to simulate where the infant’s back would be located relative to the filter. A simulated infant mannequin was not utilized in this study, because the thermal properties of a mannequin would not be representative of an infant. In particular, an infant undergoing kangaroo care is in thermal communication with his or her caregiver, making thermal modeling a challenge.

The total outdoor values for each of these parameters was taken ~2 inches above the device under direct sunlight. Measurements of UV-A radiation and irradiance were recorded for both filters from 10:04 a.m. to 7:45 p.m., with at least one measurement per hour. The measurements were recorded on 8/10/2024 in White Bear Lake, Minnesota. On that day, the sunrise occurred at 6:08 a.m. and sunset occurred at 8:25 p.m. Measurements were taken in an open space with no trees blocking the sunlight. The overall weather for the day was clear skies with occasional clouds, as discussed further in the “Results” section.

Microsoft Excel 360 was utilized for statistical analysis. A one-way ANOVA test was utilized to determine analyze the blue light irradiance values across the three different filter angles, followed by a post hoc analysis with Fisher’s least significant difference test.

## Results

**Fig. 3:**
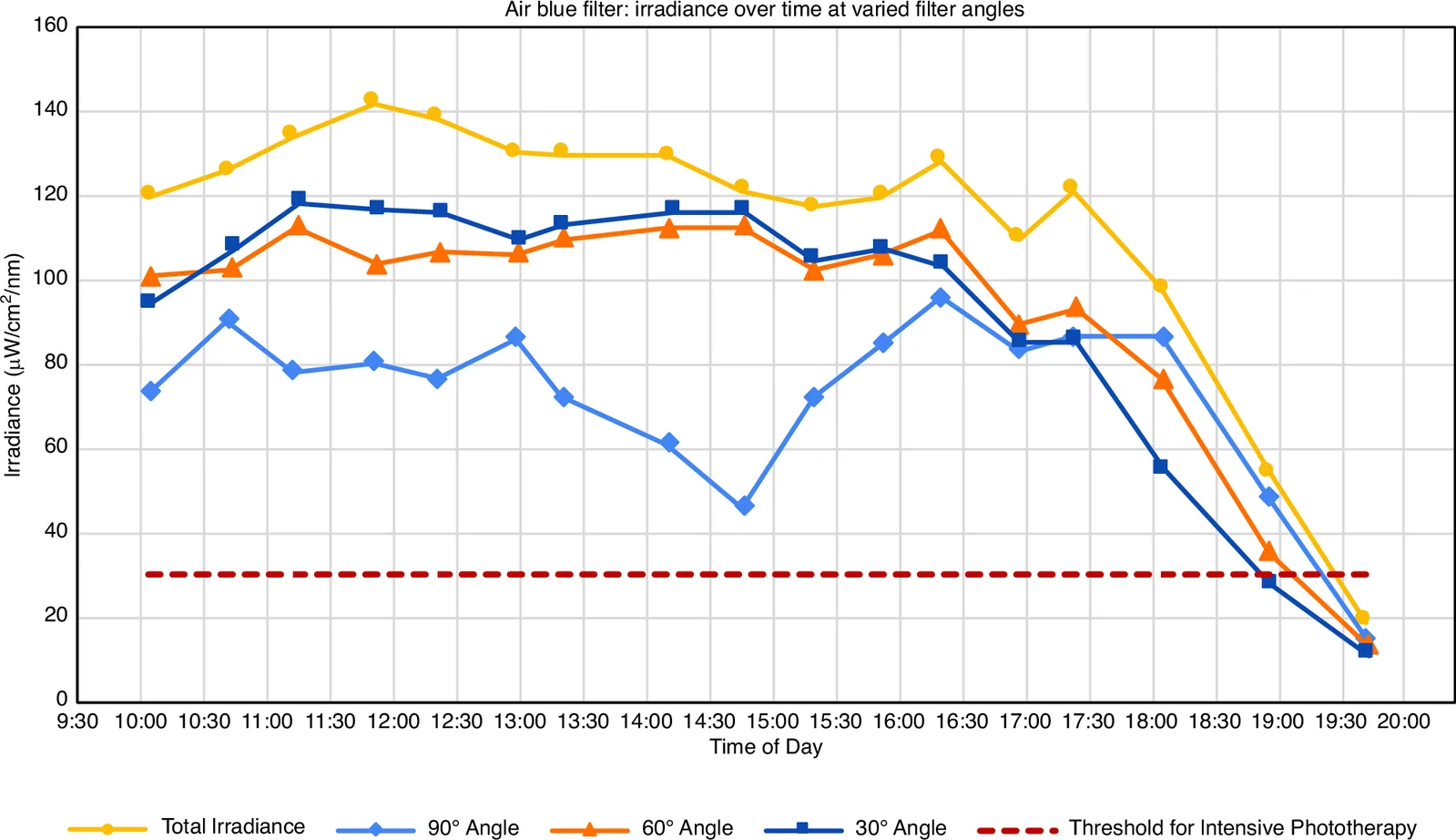
Air Blue Filter: irradiance over time at varied filter angles*.* The data demonstrates the effectiveness of the Air Blue filter for average levels of through therapeutic blue irradiance well above the threshold for intensive phototherapy.

Figure 3 shows the delivered therapeutic irradiance over time with varying angles with respect to the ground (30°, 60°, 90°) with the Air Blue filter. Over the course of the day, the following mean (standard deviation) irradiance was delivered at the following filter angles: 73.48 (19.81) μW/cm²/nm at 90°, 94.53 (27.34) μW/cm²/nm at 60°, and 94.19 (31.46) μW/cm²/nm at 30°. The one-way ANOVA test (*F*(2,51) = 3.69, *p* = 0.032) with Post-hoc Fisher’s LSD tests indicated that filter kept at a 90° had a significant difference compared to both the filters kept at the 60° (*p* = 0.012) and 30° (*p* = 0.024) angle. After 5:30, the overall total irradiance decreased as the sun began to set behind the trees. However, even at 7 pm, the irradiance values for all three filters were above the threshold for effective phototherapy, with the filters held at 60° and 90° being above the threshold for intensive phototherapy.

**Fig. 4:**
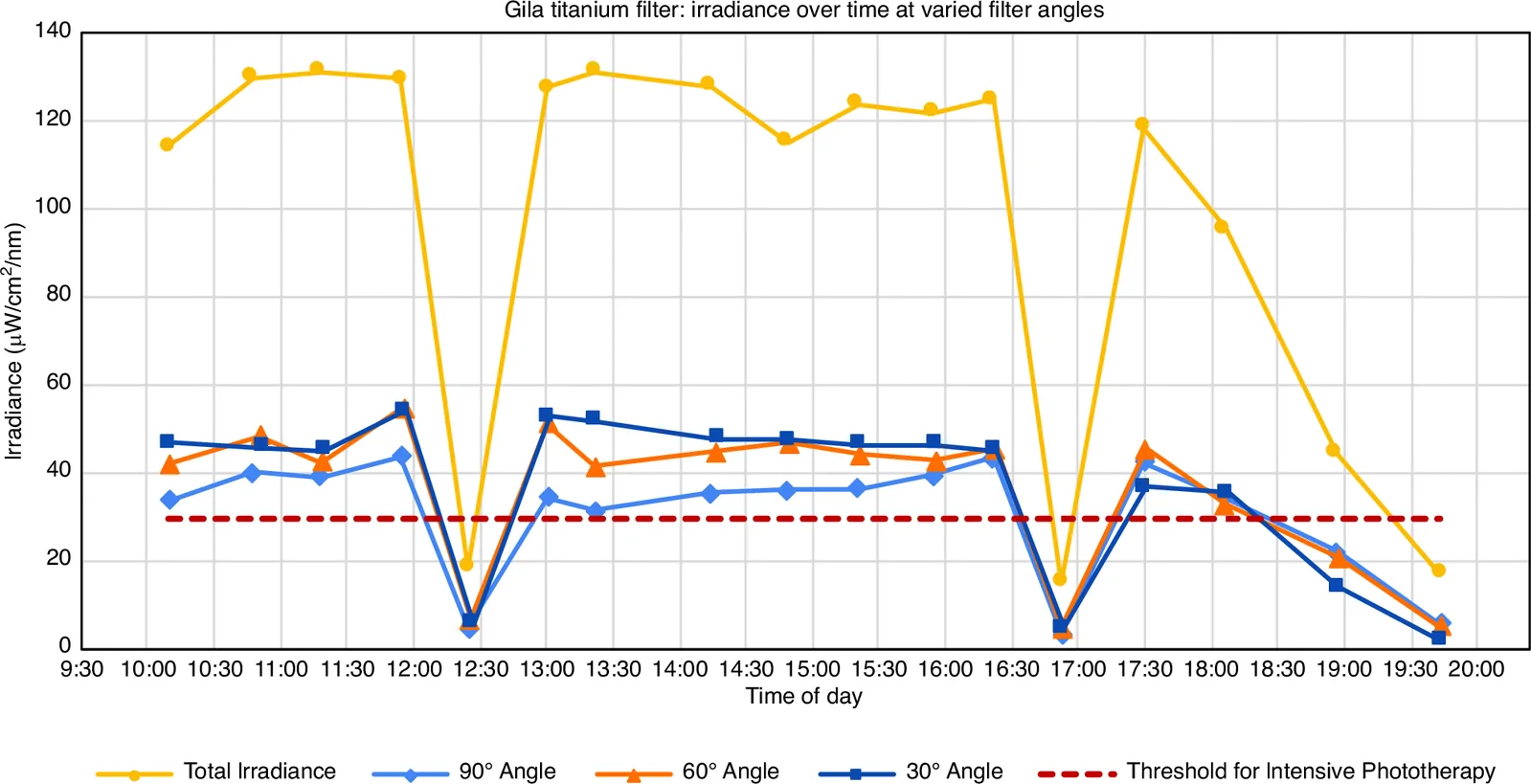
Gila Titanium Filter: irradiance over time at varied filter angles*.* The data demonstrates the effectiveness of the Gila Titanium filter throughout the data for average levels of through therapeutic blue irradiance above the threshold for intensive phototherapy.

Figure 4 shows the delivered therapeutic irradiance over time at varying angles with respect to the ground (30°, 60°, 90°) for the Gila Titanium filter. The following mean (standard deviation) values of irradiance were measured: 31.62 (13.30) μW/cm²/nm at 90°, 37.04 (16.11) μW/cm²/nm at 60°, 37.71 (17.78) μW/cm²/nm at 30°. There was no significant difference found between any of the three groups for the Gila Titanium filter, though the filter kept at 90° tended to have lower irradiance values throughout the majority of the day compared to the other two angles. The two sharp peaks downward at around 13:30 and 17:00 occurred because the two measurements for the Gila Titanium occurred when there was a large cloud blocking the sun. This reduced the overall irradiance, and as a result, the through irradiances.

Table 1 shows the external vs delivered UV-A radiation over time at varying angles with respect to the ground (30°, 60°, 90°) for both the Air Blue and Gila Titanium filters. At all three angles of the Gila Titanium filter, over 99% of harmful UV-A radiation was blocked. At all three angles of the Air Blue filter, over 99.5% of harmful UV-A radiation was blocked.

**Table 1:** For both filters, with the filters held at all three angles, on average throughout the day, over 99% of harmful UV-A radiation was blocked by the filter, indicating the safety of the device to prevent harmful irradiation passing through to the infant’s back.

Percentage of Average Harmful UV-A Radiation Blocked by Filter		
Angle of Filter	Air Blue	Gila Titanium
90°	99.73%	99.42%
60°	99.61%	99.34%
30°	99.71%	99.36%

## Discussion

### Discussion of results

The irradiance results shown in Figs. 3 and 4 demonstrate that both filters deliver therapeutic blue light for more than 5 h a day, which was the measure utilized in the randomized clinical trial conducted by Slusher et al.^6^. However, unlike previous tent/room designs, this is a mobile medical device in which the parent or caregiver could sit at different angles, hence the need to quantify delivered therapeutic blue light and blocked UV radiation at varying angles. The results demonstrate similar overall effectiveness at various angles, with all angles having an average irradiance greater than the threshold required for intensive phototherapy for most of the day. For the air blue filter, at the 90° filter angle, there was a relative decrease in delivered irradiance during the middle of the day, though it was still above the threshold for intensive phototherapy. This is likely because as the sun is overhead, it is unable to pass through the filter as well as for the 60° and 30° filter angles, but as the sun becomes closer to the horizon, a greater amount of irradiance can pass through for the 90° filter. There was a slight decrease in delivered irradiance at all angles as dusk approached. However, note that this is still above the therapeutic threshold for high intensity treatment and well above the threshold for effective phototherapy. This suggests that caregivers can sit at a wide variety of angles while carrying their infants in a phototherapy sling.

The Gila Titanium filter had less irradiance transmitted overtime compared to the Air Blue filter. In a bench study comparing multiple filter types, Vreman et al. determined that Air Blue was the ideal filter for an overcast sky when maximum blue light transmission is desired and that Gila Titanium was the ideal filter under clear skies when attenuated transmission of blue light is desirable^10^. The results from this bench study confirm these conclusions, with higher irradiance values for Air Blue filter than Gila Titanium filter, even with a plastic sheet underneath each filter.

The UV-A data shown in Table 1 demonstrates that over 99% of UV-A light was blocked with both the Gila Titanium and Air Blue filters, with a slightly higher proportion of UV-A light blocked with the Air Blue filters. As an extension, based on the bench study previously done on filtered sunlight phototherapy^10,11^, because UVA light (315–400 nm) is more likely to pass through organic filters than UVB (280–315 nm) or UVC (100–280 nm) light, it is likely that these other forms of harmful UV radiation will also be blocked^10,11^.

### Benefits of filtered sunlight phototherapy

Filtered sunlight phototherapy has been demonstrated to be low cost, non-electric, and effective, having been validated in large randomized control trials^6^. In the randomized clinical trial, filtered sunlight was found to be efficacious on 93% of treatment days that could be evaluated, compared with 90% for conventional phototherapy^6^. Filtered sunlight phototherapy also had a higher mean level of irradiance (40 vs. 17 μW per square centimeter per nanometer, *p* < 0.001)^6^. In the study, no infant met withdrawal criteria for reasons of safety or required an exchange transfusion^6^, demonstrating that filtered sunlight phototherapy is a safe treatment. However, the modes of filtered sunlight phototherapy that were tested before involved tent and room models that did not allow for easy kangaroo care. In part, this was due to difficulty with modesty and limiting parents (oftentimes the mother) with no way to do other activities of daily living if they wanted to remain with their baby while they received filtered sunlight. It additionally required additional work for the hospital staff to ensure no overheating and thus prevented kangaroo care from taking place in most infant/mother dyads. Currently there are no commercialized filtered sunlight devices.

### Benefits of kangaroo care

Continuous kangaroo care is a proven and effective method that has been shown to decrease morbidity and mortality, especially for preterm neonates. Continuous kangaroo care is strongly recommended by the World Health Organization for premature infants, especially in the first weeks of life^8^. The benefits of kangaroo care immediately after birth in all newborns include calming the neonate, reminding the child to breathe thus decreasing the frequency of apneic episodes, helping the neonate maintain normothermia, promoting bonding between the parent and neonate due to increased levels of oxytocin^8^, and helping the mother to produce more milk^9^. However, typical phototherapy devices do not often have a kangaroo care component as part of them.

The novel medical device referenced in the study combines the technologies of filtered sunlight phototherapy with kangaroo care, to enable for a non-electric, low-cost, easy-to-use medical device that has the potential to scale throughout limited-resource settings. The device also has at-home treatment potential, especially with effectiveness at multiple carrier angles.

### Limitations

Some limitations of the study include that it was not possible to assess the benefits of kangaroo care without an infant present. However, because the device is very similar to typical kangaroo care slings, it is assumed that the benefits seen with those slings such as improved caregiver/infant bonding, feeding and physiological stability would also be present in this case. Similarly, in the absence of an infant, ability to model heat transfer was limited. Air temperature was measured in front of and behind the filter, in the same manner as UV radiation and therapeutic blue radiation and similar values of air temperature were noted proximal and distal to the air filter. Note that in clinical use, the infant would absorb heat due to thermal radiation but also equilibrate to some degree with their caregiver; these bench studies were unable to simulate these thermal interactions. Further testing with measurement of infant skin temperature while they undergo treatment with filtered sunlight phototherapy and kangaroo care are warranted. The study was conducted in Minnesota, but interestingly the temperature and sun intensity on the day of the study were similar to values in Nigeria on that day. Note that on average there are significantly higher temperatures and sun intensity in Sub-Saharan Africa due to its proximity to the equator than Minnesota; bench testing in Sub-Saharan Africa could be repeated and would likely demonstrate even higher delivery of therapeutic blue light.

### Future directions

Future directions include conducting a small pilot study of feasibility in newborn infants, in which irradiance, UV-A radiation blockage, and temperature of the infant would be monitored. Ultimately, clinical trials are necessary to demonstrate a decrease in bilirubin levels for infants with jaundice treated with this device in comparison with conventional phototherapy treatments. Additionally, a usability study with infants and parents would be helpful to provide insights about overall comfortability, parent preferences, and other nuances that cannot be adequately assessed in a bench trial. Nevertheless, these bench results serve as an important first step in establishing the delivery of filtered sunlight phototherapy with kangaroo care.

## Data Availability

The datasets generated during and analyzed during the current study are available from the corresponding author on reasonable request.
